# Biomechanical analysis of clear aligners for mandibular anterior teeth intrusion and its clinical application in the design of new aligner attachment

**DOI:** 10.1186/s40510-025-00557-3

**Published:** 2025-03-10

**Authors:** Shengzhao Xiao, Caiqi Cheng, Haochen Li, Lin Li, Canao Shen, Qiping Feng, Yan Zhao, Yufeng Duan, Lunguo Xia, Fengting Chu, Bing Fang

**Affiliations:** 1 Department of Orthodontics, Shanghai Ninth People’s Hospital, Shanghai Jiao Tong University School of Medicine; College of Stomatology, Shanghai Jiao Tong University; National Center for Stomatology; National Clinical Research Center for Oral Diseases; Shanghai Key Laboratory of Stomatology; Shanghai Research Institute of Stomatology, Shanghai, 200011 China; 2 School of Health Science and Engineering, University of Shanghai for Science and Technology, Shanghai, 200093 China

**Keywords:** Clear aligner, Anterior teeth intrusion, Finite element analysis, Dentistry

## Abstract

**Background:**

During the process of intruding the mandibular anterior teeth (MAT) with clear aligners (CA), the teeth are susceptible to undesigned buccal and lingual inclinations, leading to complications such as excessive alveolar bone resorption and root exposure that significantly compromise the treatment outcome. Therefore, it is imperative to investigate the underlying causes and develop effective coping strategies.

**Methods:**

We first statistically analyzed the clinical issues, then used FEA to explore their underlying mechanisms to guide the design of attachments in clinical practice. Specifically, CBCT data before and after the intrusion treatment of MAT were collected to analyze the labial-lingual inclination of the MAT and the distance between the root apex and alveolar bone wall. Finite element analysis (FEA) models of MAT undergoing vertical intrusion with standard CA were created with eight incisor mandibular plane angles (IMPA) to assess displacement trends, labial and lingual moments, and crown contact forces. Additionally, six aligner attachments were designed to simulate and analyze their biomechanical mechanisms.

**Results:**

Significant differences were observed in changes before and after treatment. When the IMPA was 90°, the crown experienced a labial moment. The labial root control ridge (RCR) increased the labial moment of the crown, while the lingual RCR and labial attachment (LA) increased the lingual moment. The lingual fossa excavating holes (LFEH) group also increased the labial moment. The lingual RCR enhanced the lingual movement of the crown, whereas the LFEH promoted labial movement. During the intrusion of MAT, a comprehensive design incorporating labial intrusive attachments, labial RCR, lingual RCR, and LFEH can be employed to ensure true vertical intrusion of the lower anterior teeth.

**Conclusion:**

This study revealed the biomechanical changes during intrusion, and innovatively designed the LFEH, thereby promoting the development of novel orthodontic techniques and improving clinical treatment outcomes.

## Introduction

Anterior teeth intrusion (ATI) refers to the process of pressing the teeth into the alveolar bone to reduce their exposure in the oral cavity [1]. It is a common technique in orthodontic treatment, particularly for addressing deep overbite of the anterior teeth. Deep overbite is a frequent dental and occlusal characteristic of malocclusion, primarily caused by abnormal vertical development of the maxillary and mandibular dental arches and jaws. The clinical manifestations include excessive eruption of teeth and overdevelopment of the alveolar bone in the maxillary anterior region, or insufficient eruption and underdevelopment of the alveolar bone in the mandibular posterior region. Normal or excessive maxillary development is often accompanied by underdevelopment of the mandible. Under the influence of the ascending jaw muscles, the mandible rotates counterclockwise, leading to a reduced mandibular plane angle, which presents as a deep overbite. Deep overbite is closely linked to oral function and health. If left untreated, it can lead to traumatic gingivitis, periodontitis, alveolar bone resorption, and may also result in temporomandibular joint disorders [1, 2]. Correcting deep overbite is often a crucial step in managing complex cases. Therefore, addressing deep overbite is of great importance for both the health and aesthetics of patients.

Additionally, ATI is a common technique in the orthodontic treatment of Class II malocclusion [3, 4]. Class II malocclusion is often associated with symptoms such as a convex facial profile, mandibular retrusion, deep overbite, deep overjet, and an excessively deep Spee curve, leading to facial aesthetic imbalance and negatively impacting both physical and mental health. In clinical practice, leveling the Spee curve is a priority. A normal Spee curve provides optimal conditions for retracting the maxillary anterior teeth, thereby restoring facial aesthetics [5]. The alignment of the Spee curve directly influences the automatic rotation of the mandible and the aesthetic facial proportions. Thus, ATI is an important step in treating Class II malocclusion [3, 4, 6, 7].

Intrusion of the mandibular anterior teeth (MAT) is an important step for the treatment of deep overbite and Class II malocclusion [8, 9]. Based on the direction of intrusion, ATI is generally classified into labial tilt intrusion, lingual tilt intrusion, and true vertical intrusion. Clinically, true vertical intrusion is most often required, which refers to the teeth being pressed into the alveolar bone without labial or lingual deviation [10]. In traditional fixed bracket orthodontic treatment, techniques such as the rocking-chair archwire, multi-loop edgewise, or micro-implant anchorage are often employed to intrude the anterior teeth [4, 11, 12]. These methods apply force to the labial brackets, which in turn applies force to the labial side of the teeth, with most cases resulting in labial-inclined intrusion [13, 14]. Clear aligners (CA), made from specialized plastic through a hot pressing process, are personalized, easy to clean, aesthetically pleasing, and removable, and have become widely used in orthodontic treatment [15]. Due to its enveloping loading mode and the fact that the direction of the applied force is closer to the tooth’s long axis, CA theoretically make it easier to achieve true vertical intrusion (i.e., the tooth is not intruded obliquely along its long axis) [16, 17]. Intrusion that deviates from the intended direction is referred to as undesigned buccal and lingual inclination, indicating the occurrence of unexpected labial and lingual movements of the incisors throughout orthodontic treatment. These movements can lead to an excessive inclination towards either the lingual or labial side, thereby heightening the likelihood of root exposure on the respective side. Such an outcome is deemed adverse and diverges from the anticipated benefits of orthodontic care. Studies have shown that the efficiency of intrusion with CA ranges from 18.3 to 79%, with anterior teeth being intruded by 0.72 to 2.10 mm [18–21].

During ATI, if the patient’s alveolar bone condition is poor or the direction of tooth intrusion is not well controlled, undesigned labial or lingual intrusion may occur, increasing the risk of labial or lingual bone fenestration and dehiscence [22]. Bone dehiscence refers to a V-shaped bone defect in the alveolar bone on the buccal or lingual/palatal sides of the teeth, exposing up to one-third of the root near the neck. Alveolar bone defects that do not extend to the alveolar ridge crest are referred to as bone fenestration [23, 24]. Since aligners move teeth through mechanical forces, studying the force mechanisms of aligners on teeth is crucial.

Finite element analysis (FEA) is an effective method for studying biomechanical forces. It can model the forces applied by aligners on teeth and predict their effects, making it a valuable tool for guiding clinical aligner applications [25]. FEA can accurately simulate and analyze the stress and displacement changes in the crown, root, periodontal ligament, and other tissues after orthodontic force is applied [26]. However, there are currently few biomechanical studies focused on the mechanics involved in CA treatment. No established framework exists for understanding undesigned buccal and lingual inclinations patterns or their biomechanical mechanisms, which is crucial for improving the design of new aligner attachments, enhancing clinical outcomes, and providing a scientific basis for controlling mandibular ATI.

Our previous study observed a higher incidence of bone fenestration in the anterior teeth region when clear aligners were used to effectively intrude the anterior teeth, with most cases occurring on the labial side [22]. In this study, we aimed to further investigate the biomechanical mechanism underlying this phenomenon. This study was structured in two phases: the first phase involved a CBCT assessment to identify key issues, and the second phase utilized finite element analysis to evaluate the effectiveness of potential solutions based on the initial findings. This study explored the optimal combination of attachment designs for lower anterior teeth with different degrees of labial inclination, in order to prevent undesigned buccal and lingual inclinations variations that occur during the clinical process with CA for mandibular ATI.

### Materials and methods

In this study, we first statistically analyzed the issues identified in clinical practice, then used FEA to explore the underlying mechanisms, and finally applied these findings to guide the design of attachments in clinical practice. Specifically, we first conducted a statistical analysis of changes in tooth inclination and the distance between the tooth root apex and the alveolar bone wall during anterior teeth intrusion treatment. Next, we designed and constructed a three-dimensional finite element simulation model to analyze the labial and lingual movement of the tooth root during intrusion at different mandibular incisor inclination angles under the influence of clear aligners. Finally, six attachment designs were created. Finite element simulation was used to compare and analyze the orthodontic effects of these different attachment designs, providing guidance for clear aligner design in mandibular anterior teeth intrusion. This helps prevent undesigned buccal and lingual inclinations during the intrusion process.

### Statistical analysis of changes in the MAT angle and alveolar bone wall thickness before and after orthodontic treatment

CBCT images of 30 patients (male-to-female ratio of 1:1, aged 18–25 years) from the Department of Orthodontics, Ninth People’s Hospital, affiliated with Shanghai Jiao Tong University School of Medicine, were collected before and after CA intrusion treatment. The vertical intrusion volume, inclination angle, and alveolar bone wall (ABW) thickness of the left mandibular central incisors (31 tooth) were measured both pre- and post-treatment. The inclination angle was defined as the angle between the long axis of the mandibular central incisor and the palatal plane (IPPA, Incisor to Palatal Plane Angle). ABW thickness was measured as the vertical distance from the long axis of the mandibular central incisor to the buccal and lingual ABW. Statistical analysis was performed using IBM SPSS Statistics (version 26; IBM Corp., Armonk, NY, USA). Paired sample T-tests were conducted to assess the significance of changes in inclination angle, lingual ABW thickness, and labial ABW thickness before and after treatment.

### Establishment of a three-dimensional FEA model for mandibular ATI using CA

FEA is a numerical computation method used to predict how structures react under physical forces. This method involves breaking down complex structures into many smaller, simpler parts known as finite elements. Mathematical models are then used to analyze the behavior of each part, and the results are aggregated to predict the behavior of the entire structure. Initially, we acquire three-dimensional structures like teeth using CT scans. Then, we discretize these complex structures into numerous smaller, simpler finite elements through the process of finite element meshing. Following this, we assign material properties to the model, define the loading conditions, and conduct calculations to derive the results.

The standard mandibular model (i21D-400 C, Nissin Dental Products Inc., Tokyo, Japan) was scanned using industrial CT (METROTOM 1500, Carl Zeiss AG, Oberkochen, Germany) with a scanning layer thickness of 0.1 mm. The resulting images were imported into Mimics 19.0 (Materialise Inc., Leuven, Belgium) for three-dimensional reconstruction, which involved grayscale thresholding, region growing, and 3D calculation to obtain the three-dimensional morphology of the teeth. The resulting three-dimensional model was then imported into Geomagic Studio 12 (Raindrop Geomagic Inc., Research Triangle Park, NC, USA), where operations such as mesh doctor inspection, relaxation, spike removal, filling, contour line editing, and grid construction were performed sequentially. After these operations, the three-dimensional model data of the teeth was exported and saved [25, 26]. HyperMesh 14.0 (Altair Engineering Inc., Troy, MI, USA) was used for meshing, with a uniform extension of 0.3 mm outward along the root surface to obtain a simplified periodontal membrane morphology model [22, 27]. Atreat Designer software (Time Angels Medical Device Co., Ltd., Shanghai, China) was used for digital orthodontic design to manage and develop the orthodontic treatment plan. Atreat Manufacture software (Time Angels Medical Device Co., Ltd., Shanghai, China) was employed to automatically fill the interdental space structure of the aligner, while the aligner model was generated using the hot-press molding process simulation module in MasterForce software (Time Angels Medical Device Co., Ltd., Shanghai, China). Based on the actual clinical situation, a three-dimensional model of the teeth, periodontal ligament, and CA was established according to predefined combination conditions, creating multiple assembly models, which were then imported into MasterForce software. Atreat Processor software was used to define the coordinates of the initial model, repair and segment the tooth surface, and generate a 3D morphological model of the tooth. Finally, a three-dimensional FEA model of the CA worn on the teeth was created.

### Definition of material parameters and meshing in the FEA model

The material properties of the teeth, periodontal ligament, and aligner were set as homogeneous and isotropic [25]. The elastic modulus was set to 20,600 MPa, 0.689 MPa, and 2,000 MPa, with Poisson’s ratios of 0.30, 0.49, and 0.30, respectively [28]. The periodontal ligament was modeled using hexahedral solid elements, the aligner with triangular shell elements, and the rest of the model with tetrahedral solid elements. The mesh size was determined through convergence analysis, increasing mesh density until the deviation in estimated stress was less than 5%. The final mesh density was set to 0.3 mm, with the model divided into 747,183 elements and 981,581 nodes.

### Boundary condition settings and description of the calculation method

The contact between the teeth and CA, as well as between the teeth and periodontal ligament, was defined as face-to-face contact, with the outer side of the periodontal ligament set as a binding constraint [29]. Abaqus 2018 (Simulia Inc., Providence, RI, USA) was used to iteratively calculate the equilibrium state at each moment during the tooth displacement caused by contact forces. The process continued until the entire system reached equilibrium, outputting the force and moment of the tooth in the labiolingual direction.

### FEA condition design

Seven groups of CA conditions were established, including conventional, labial intrusion attachment (LIA), labial root control ridge (RCR), lingual RCR, lingual fossa excavating holes (LFEH), LFEH with labial attachment (LA) combination, and LFEH with labial RCR combination. The incisor mandibular plane angle (IMPA) was set at 75°, 80°, 85°, 90°, 95°, 100°, 105°, and 110°. The left mandibular central incisor was designated as the center of resistance, located two-fifths from the alveolar ridge crest and three-fifths from the root apex [22]. Changes in the moment and displacement of the mandibular anterior tooth crown were analyzed, along with the Von Mises stress and contact force on the aligner near the left and right mandibular central incisors.

## Results

### Analysis of the angle changes during ATI with CA and ABW thickness

Based on CBCT image data before and after CA intrusion treatment, the intrusion amount, inclination angle, and ABW thickness of the MAT were measured, followed by statistical analysis to assess significance. The results showed a significant change in tooth inclination before and after treatment.

**Fig. 1:**
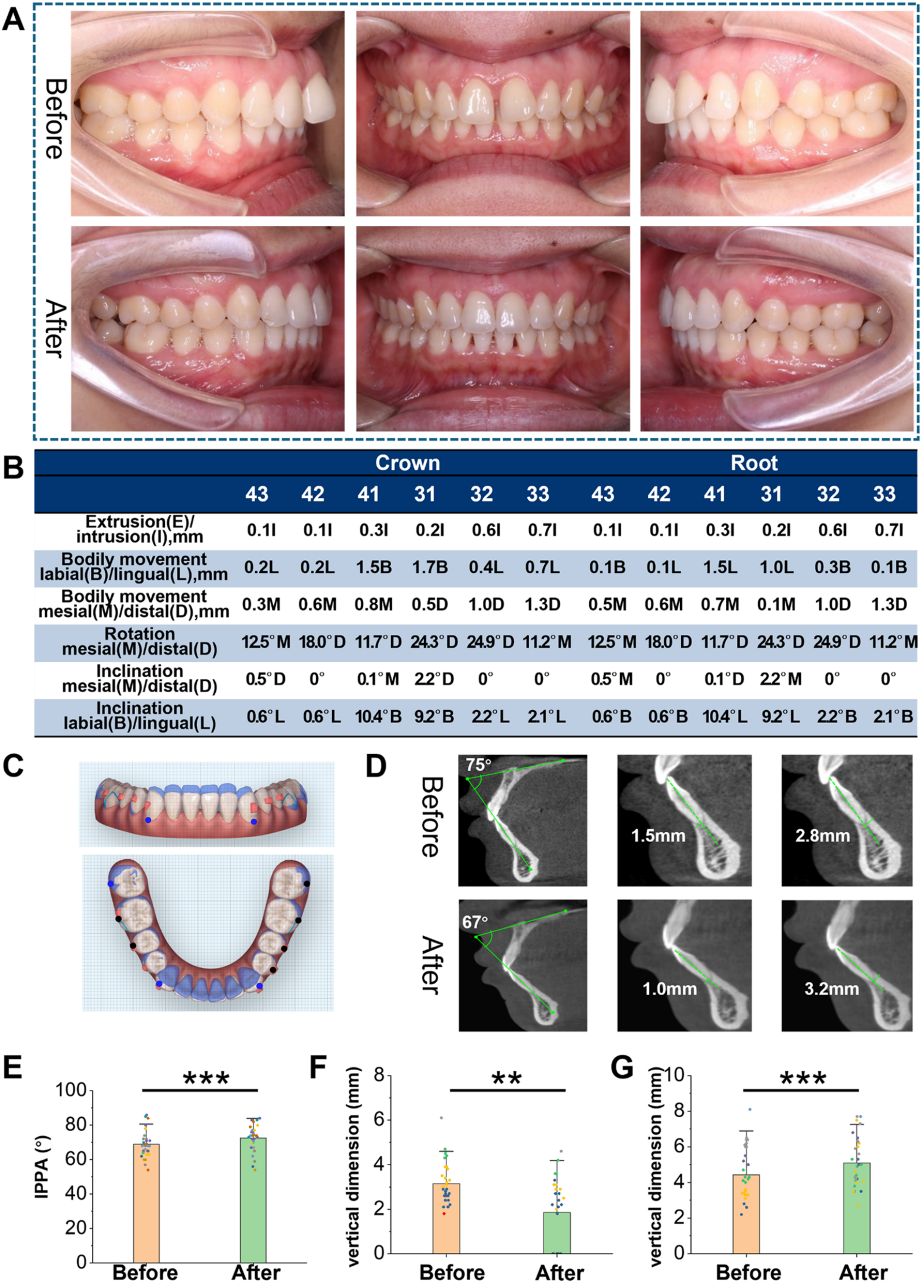
Changes in the inclination of left MAT (31 teeth) and the thickness of lingual alveolar bone before and after CA treatment. (**A**) Intraoral images of the patient before and after CA treatment show significant intrusion of the MAT. (**B**) Tooth movement data of the MAT treated with CA. The movement data, from top to bottom, include extrusion (**E**) or intrusion (**I**), labial movement (**B**) or lingual movement (**L**), mesial movement (**M**) or distal movement (**D**), mesial rotation (**M**) or distal rotation (**D**), mesial inclination (M) or distal inclination (**D**), and labial inclination (**B**) or lingual inclination (**L**). (**C**) Simulation of tooth movement in CA simulation software. The blue represents the tooth before treatment, and the white represents the tooth after treatment. (**D**) Changes in the inclination angle and thickness of the ABW were measured before and after treatment. The angle between the maxillary palatal plane and the long axis of the tooth was defined as the inclination angle of the mandibular incisor (IPPA), and the thickness of the ABW was defined as the vertical distance from the long axis of the mandibular incisor to the labial and lingual ABW. (**E**) The changes in inclination angle before and after treatment were statistically analyzed. The inclination angle of the mandibular incisors before treatment was less than 90 degrees, with an average of 68.93°, and the average IPPA after treatment was 72.53° (*P* < 0.001). (**F**) Statistical analysis of changes in the vertical distance between the root and the lingual ABW before and after treatment. The vertical distance between the long axis of the root and the lingual ABW was 4.417 mm before treatment and increased to 5.093 mm after treatment (*P* < 0.01). (**G**) Statistical analysis of changes in the vertical distance between the root and the labial ABW before and after treatment. The vertical distance between the long axis of the root and the labial ABW was 3.153 mm before treatment and decreased to 1.850 mm after treatment (*P* < 0.001)

**Fig. 2:**
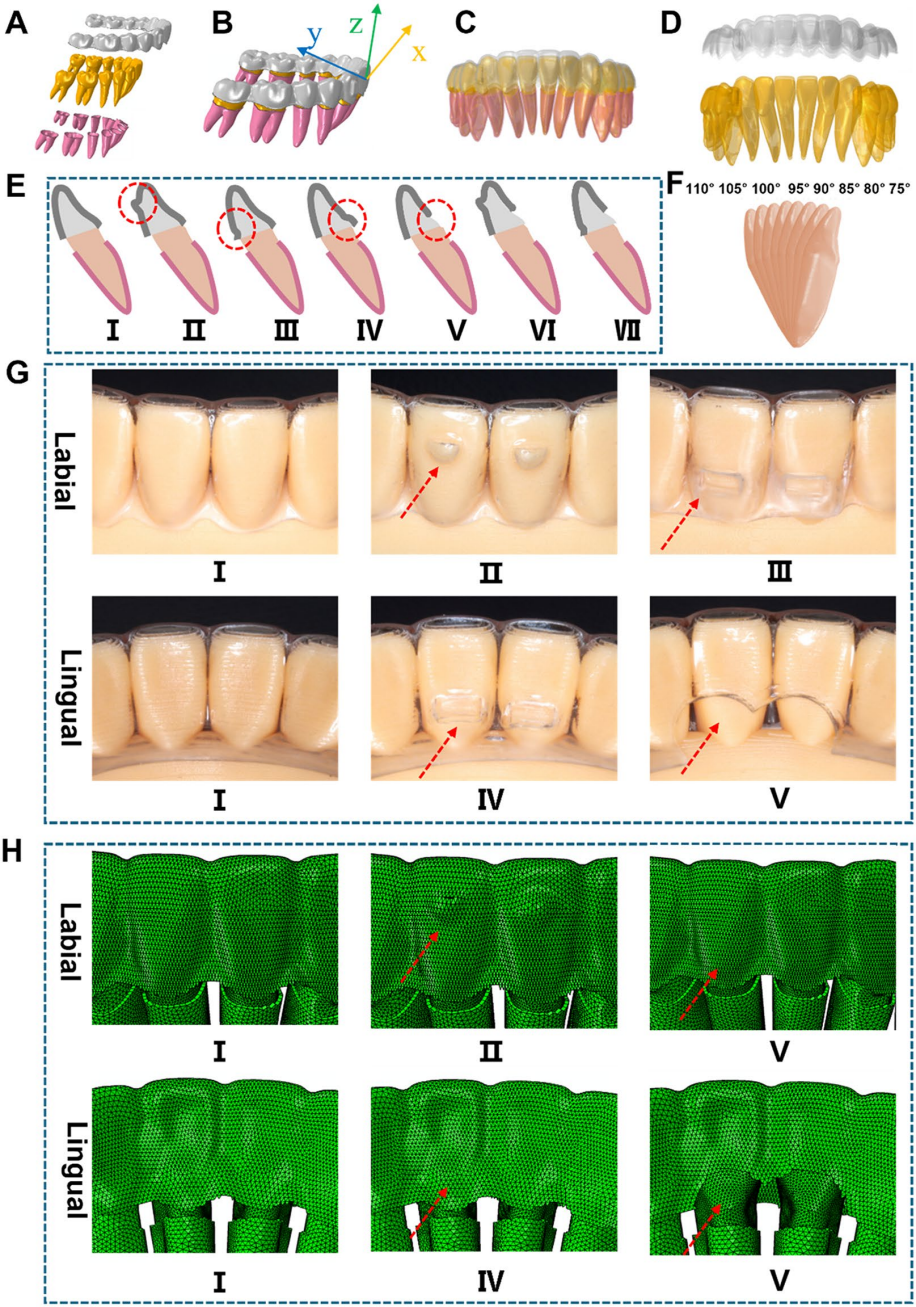
Establishment of a FEA model for mandibular ATI using CA. (**A**) Construction of a three-dimensional model of the aligners, teeth, and periodontal ligament. (**B**) Establishment of a local coordinate system with the proximal-distal direction of the teeth as the X-axis and the labial-lingual direction as the Y-axis. (**C**) Setting up a FEA model of the MAT with a 0.2-mm intrusion. (**D**) Demonstration of the effects of aligners and tooth intrusion, where the lighter color represents the position before intrusion and the darker color represents the position after the 0.2-mm intrusion. (**E**) The left mandibular central incisor aligners were divided into the following groups: conventional (I), LIA (II), labial RCR (III), lingual RCR (IV), LFEH (V), LFEH combined with LA (VI), and LFEH combined with labial RCR (VII). (**F**) Models were set up with different tooth inclination angles, including 110°, 105°, 100°, 95°, 90°, 85°, 80°, and 75°. (**G**) Physical images of labial and lingual attachments in the anterior region of CA: conventional group (I), LIA group (II), labial RCR group (III), lingual RCR group (IV), and LFEH group (V). (**H**) FEA model design results of labial and lingual attachments in the anterior region of CA: conventional group (I), LIA group (II), labial RCR group (III), lingual RCR group (IV), and LFEH group (V)

Fig. 1 A shows the left, front, and right views of the patient’s mouth before and after CA treatment, with significant intrusion of the MAT. Figure 1B presents the movement data of the crown and root positions of the anterior teeth after treatment. Figure 1C shows the simulation of tooth movement in the CA simulation software. The blue represents the teeth before treatment, and the white represents the teeth after treatment. Figure 2D illustrates the measurements of the inclination angle and ABW thickness between the long axis of the mandibular central incisor and the labial and lingual sides before and after treatment. The angle between the maxillary palatal plane and the long axis of the tooth was defined as the inclination angle of the mandibular incisor (IPPA, Incisor to Palatal Plane Angle). The thickness of the ABW was defined as the vertical distance from the long axis of the mandibular incisor to the labial and lingual ABW. A paired t-test was used to analyze changes in the inclination angle before and after treatment. The inclination angle of the mandibular incisors before treatment was less than 90 degrees, with an average of 68.93°. After treatment, the average IPPA decreased to 72.53° (*P* < 0.001) (Fig. 1E). A statistical analysis of changes in the thickness of the lateral ABW before and after treatment showed that the vertical distance between the long axis of the root and the labial ABW was 3.153 mm before treatment, decreasing to 1.850 mm after treatment (*P* < 0.01) (Fig. 1F). Statistical analysis was conducted on the changes in the thickness of the medial ABW before and after treatment. The vertical distance between the long axis of the root and the lingual ABW was 4.417 mm before treatment, decreasing to 5.093 mm after treatment (*P* < 0.001) (Fig. 1G)

### Establishment of a three-dimensional FEA model for the overall intrusion of anterior teeth using CA

After scanning the standard mandibular model using industrial CT, the images were imported into Mimics and processed with HyperMesh. Atreat Designer was used for digital orthodontic design, while Atreat Manufacture automatically filled the interdental space of the aligner. The hot-press molding process simulation module in MasterForce was then used to calculate and generate the aligner model. Finally, a three-dimensional FEA model was created, including the aligners, teeth, and periodontal ligament (Fig. 2A). The long axis of the tooth was determined based on its anatomical morphology, with the direction of the long axis defined as the z-axis. The boundary between the crown and the gingiva forms a curved surface, and the intersection of this surface with the z-axis was set as the origin of the coordinate system. The mesial and distal surfaces were also determined based on the tooth’s anatomical morphology, with the mesial and distal directions aligned along the x-axis. Following the perpendicular relationship of the coordinate axes in the rectangular coordinate system, the y-axis (representing the buccal and lingual directions) was defined (Fig. 2B). The symmetrical center line along the jaw profile resulted in the intrusion of the left mandibular central incisor by 0.2 mm (Fig. 2C and D).

Seven groups of working conditions were established: conventional (Fig. 2E-I, G-I and H-I), LIA (Fig. 2E-II, Fig. 2G-II and Fig. 2H-II), labial RCR (Fig. 2E-III, Fig. 2G-III and Fig. 2H-III), lingual RCR (Fig. 2E-IV, Fig. 2G-IV and Fig. 2H-IV), LFEH (Fig. 2E-V, G-V and H-V), combination of LFEH and LA (Fig. 2E- VI), and combination of LFEH and labial RCR (Fig. 2E-VII). The designed incisor mandibular plane angles (IMPA) were set at 75°, 80°, 85°, 90°, 95°, 100°, 105°, and 110°, respectively (Fig. 2F). Physical results of labial and lingual attachment designs in the anterior region for CA (Fig. 2G) and FEA model design results (Fig. 2H).

### Moment analysis of intruded MAT with CA

**Fig. 3:**
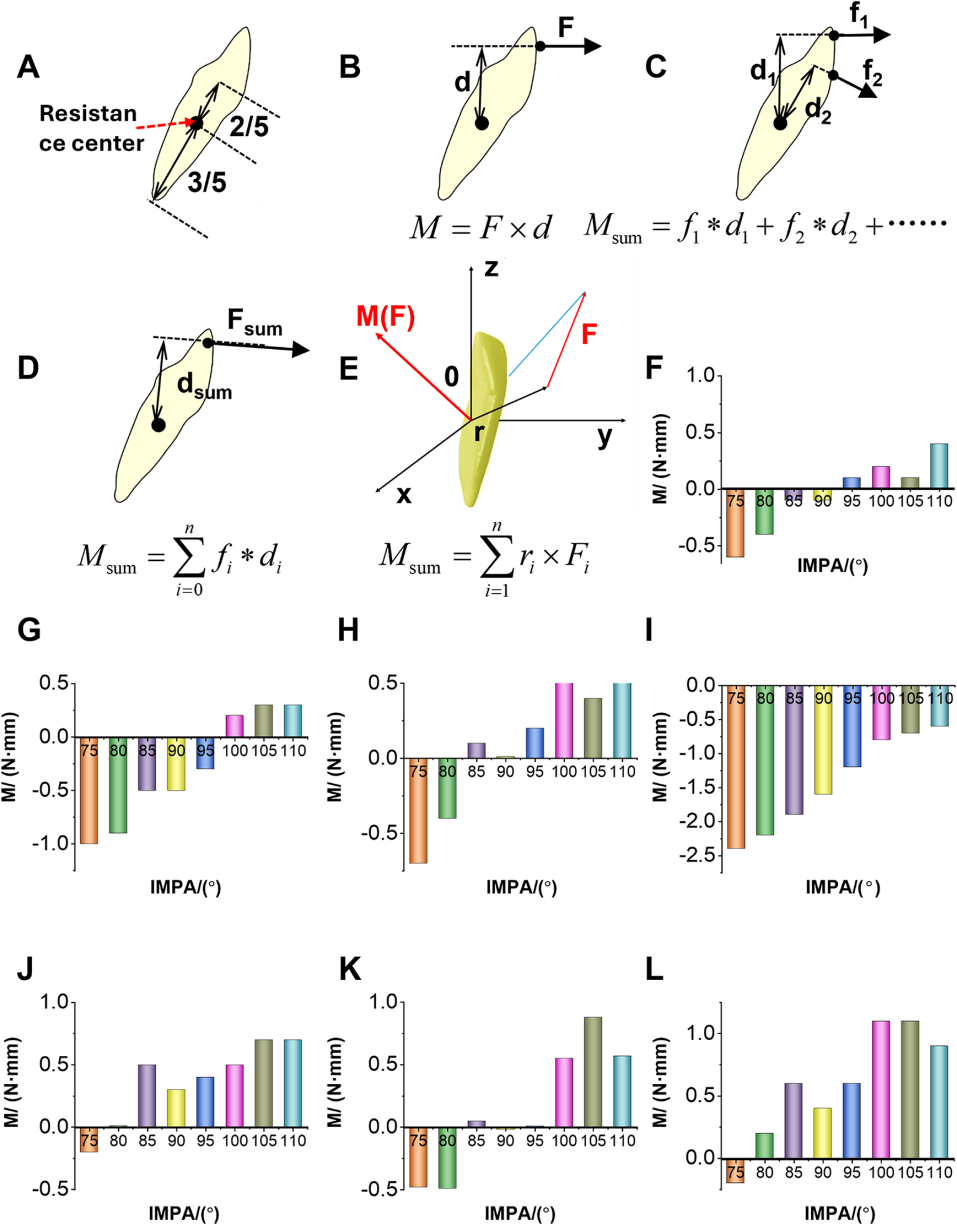
Moment analysis of MAT intruded by CA. (**A**) Diagram of the center of resistance, located two-fifths of the distance from the alveolar ridge crest and three-fifths from the root apex. (**B**) Using equation $\:M=F\times\:d$, the moment is calculated. (**C**) The force and force arm acting on each point of the two-dimensional tooth surface are determined using equation ${M_{{\text{sum}}}}={f_1} * {d_1}+{f_2} * {d_2}+ \ldots \ldots$. (**D**) The total force-distance product on the two-dimensional tooth surface is calculated using equation $\:{M}_{\text{sum}}={\sum\:}_{i=1}^{n}{f}_{i}\times\:{d}_{i}$. (**E**) The three-dimensional moment analysis diagram: r represents the vector magnitude, $\:M\left(F\right)$ represents the moment vector, and $\mathop M\nolimits_{{{\text{sum}}}}^{ * } =\sum\limits_{{i=1}}^{n} {{f_i} \times {d_i}}$ represents the three-dimensional moment result. Conventional group (**F**), LIA group (**G**), labial RCR group (**H**), lingualRCR group (**I**), LFEH group (**J**), combination of LFEH and LA group (**K**), and combination of LFEH and labial RCR group (**L**). Note: A positive moment indicates the labial direction, while a negative moment indicates the lingual direction

The left mandibular central incisor was positioned with two-fifths of its length from the alveolar ridge crest and three-fifths from the root apex as the center of resistance [22], as indicated by the red arrow in Fig. 3A.

Under two-dimensional conditions, the moment is calculated using formula$\:M=F\times\:d$, where F is the force applied at a specific point on the tooth, and d is the corresponding force arm. The moment at each point on the crown surface is then calculated $\:{M}_{\text{sum}}={f}_{1}*{d}_{1}+{f}_{2}*{d}_{2}+\dots\:\dots\:$, and the total moment for the entire tooth surface is calculated ${M_{{\text{sum}}}}$, yielding the final result $\:{M}_{\text{sum}}={\sum\:}_{i=1}^{n}{f}_{i}\times\:{d}_{i}$, as shown in Fig. 3B and D.

In practical applications, the calculation of the total moment for the three-dimensional structure is illustrated in Fig. 3E. The moment of a force about the center of resistance is equal to the cross product of the vector from the moment center to the point of force application and the force itself. The analytical expressions for these vectors are represented as $\:r=xi+yj+zk$ and $\:F={F}_{X}i+{F}_{Y}j+{F}_{Z}k$, respectively. Therefore:

$$\begin{gathered} M(F) = r \times F \hfill \\= \left( {\begin{array}{*{20}{c}} i&j&k \\ x&y&z \\ {{F_x}}&{{F_y}}&{{F_z}} \end{array}} \right) \hfill \\= (y{F_z} - z{F_y})i + (z{F_X} - x{F_z})j + (x{F_y} - y{F_x})k \hfill \\ \end{gathered} $$

where i, j, and k are the unit vectors along the x, y and z axes, respectively. From this we can determine the moment as shown in $\mathop M\nolimits_{{{\text{sum}}}}^{ * } =\sum\limits_{{i=1}}^{n} {{f_i} \times {d_i}}$ [30].

The results of the labiolingual moment analysis of the anterior tooth crown under different labial inclinations are shown in Fig. 3F-L. In the conventional group, where no auxiliary structures were used, upright teeth (IMPA = 90°, 95°) exhibited minimal labiolingual moments during the intrusion process. In contrast, labially inclined teeth (IMPA = 100°, 105°, 110°) experienced significant labial moments during intrusion, while lingually inclined teeth (IMPA = 70°, 80°, 85°) exhibited significant lingual moments. The more pronounced the labial or lingual inclination of the teeth, the more evident this trend became (Fig. 3F).

The addition of LIA increased the tendency of the crown to move lingually. Teeth with IMPA values of 75°, 80°, 85°, and 90° still experienced a lingual moment during intrusion, but the magnitude was greater than that of the conventional group. In the 95° group, the moment shifted from a labial moment in the conventional group to a lingual moment. However, in the groups with severe labial inclination (105° and 110°), the lingual moment did not change significantly with the addition of the LIA (Fig. 3G).

The addition of labial RCR increased the tendency of the teeth to move labially, particularly in the severe labial inclination group (IMPA = 85°, 90°, 95°, 100°, 105°, 110°). These teeth experienced an increased labial moment during intrusion. In contrast, in the severe lingual inclination group (IMPA = 75°, 80°), the addition of labial RCR increased the lingual moment (Fig. 3H).

The addition of lingualRCR increased the lingual movement tendency of the crown, with a more pronounced effect than that of the LIA group (Fig. 3I). Teeth with IMPA values of 95°, 100°, 105°, and 110° experienced a lingual moment during intrusion, which was opposite to the force direction in the conventional group. Teeth with IMPA values of 75°, 80°, 85°, and 90° also experienced a lingual moment during intrusion, but the moment was significantly higher than in the conventional group, with a more noticeable increase compared to the LIA group. In contrast, the LFEH group increased the labial moment of the teeth compared to the conventional group (Fig. 3J).

The combination of LFEH and labial RCR significantly increased the labial moment of the teeth compared to the LFEH group alone. The labial moment was especially increased in the severe lingual inclination group (IMPA = 75°, 80°), compared to the simple labial RCR group (Fig. 3K). Additionally, the combination of LFEH and LA significantly reduced the lingual moment of the teeth compared to the LFEH group alone. However, in the severe labial inclination group (IMPA = 105°, 110°), the lingual moment showed no significant change (Fig. 3L).

### Analysis of displacement changes in the MAT of with CA

**Fig. 4:**
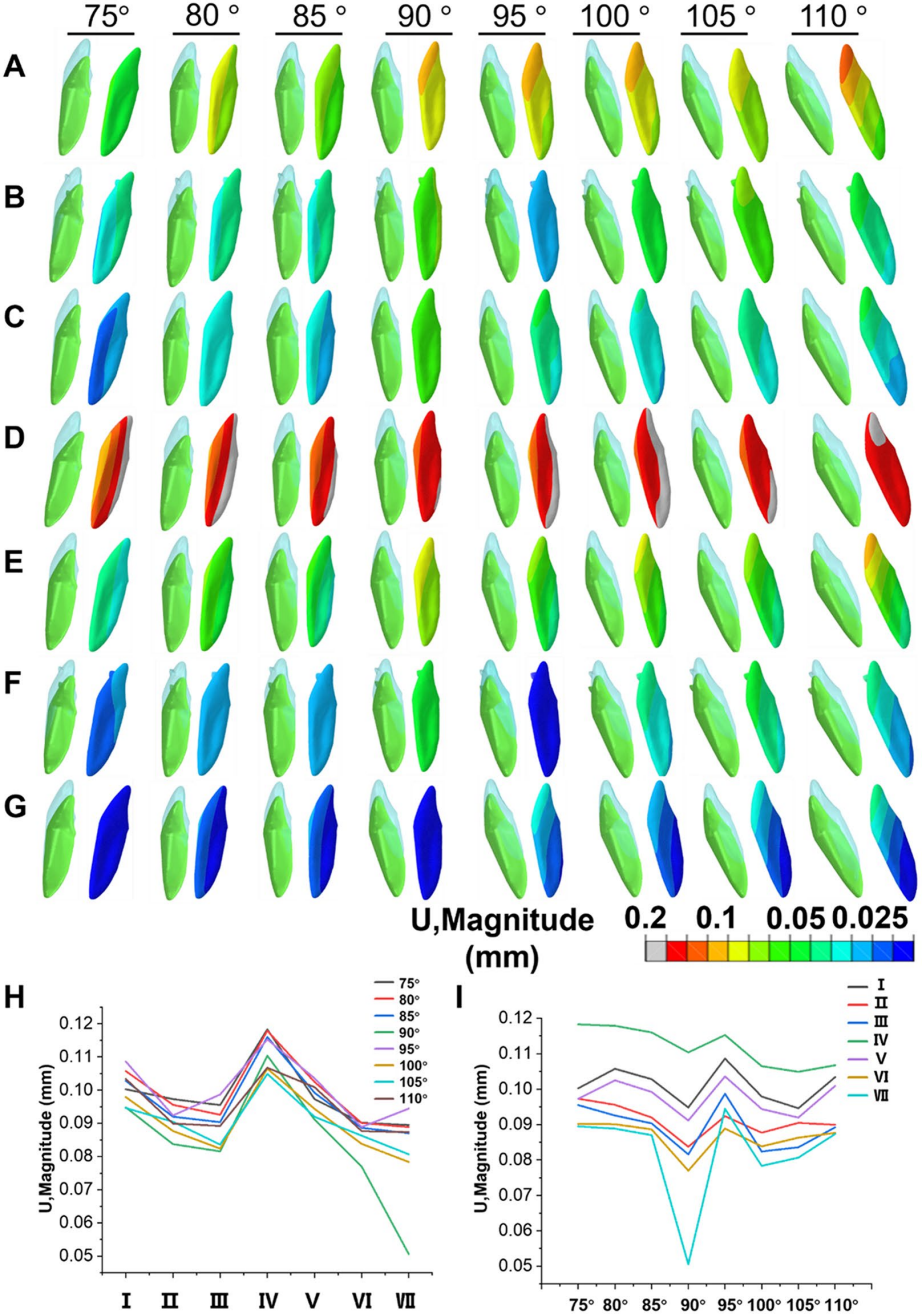
Analysis of Displacement Trends in MAT During Intrusion. **A**-**G** represent the tooth displacement maps before and after mandibular ATI: Conventional group (**A**), LIA group (**B**), labial RCR group (**C**), lingual RCR group (**D**), LFEH group (**E**), combination of LFEH and LA group (**F**), and combination of LFEH and labial RCR group (**G**). Blue indicates the position before intrusion, and green indicates the position after intrusion. The displacement change is magnified by 50 times. The maximum value is shown in the statistical displacement cloud diagram, with (**H**) representing tooth displacement at different angles under the same condition, and (**I**) representing tooth displacement under different conditions at the same angle. The different groups are represented as follows: conventional (I), LIA (II), labial RCR (III), lingual RCR (IV), LFEH (V), LFEH combined with LA (VI), and LFEH combined with labial RCR (VII). The color bar represents the magnitude of displacement, with red indicating large displacement and blue indicating small displacement

After the intrusion of the MAT, the displacement cloud diagram and statistical chart showing the labial and lingual displacement changes of the left mandibular central incisors in each group are presented in Fig. 4. The results indicated that lingual displacement was prominent when the IMPA was 75°, 80°, and 85°. In contrast, labial displacement was more pronounced when the IMPA was 100°, 105°, and 110°. The lingual control root ridge group exhibited the largest displacement, while the LFEH group combined with the LA and the LFEH combined with the labial RCR showed less displacement compared to the LFEH group.

The results in Fig. 4A show that after the intrusion of the MAT, the conventional group exhibited the largest lingual movement when the IMPA was 75°, and the largest labial movement when the IMPA was 110°. There was no significant labial or lingual displacement change when the IMPA was 90°. The addition of LIA increased the tendency of the crown to move lingually (Fig. 4B), while the addition of labial RCR increased the tendency of the teeth to move labially (Fig. 4C). The addition of lingual attachments increased the tendency for crown movement to the lingual side and enhanced the tendency for lingual inclination more than LA (Fig. 4D). The maximum displacement at the same angle for different attachments was measured, revealing that the lingual RCR group had the largest displacement, while the overall displacement in the LFEH and labial RCR group was significantly lower than that of the other groups (Fig. 4H). The maximum displacement for the same attachment at different angles was also measured, showing that the combination of LFEH and labial RCR was the most sensitive to angle changes (Fig. 4I).

### The mechanical analysis of CA intruding into the middle and anterior regions of the mandibular teeth

**Fig. 5:**
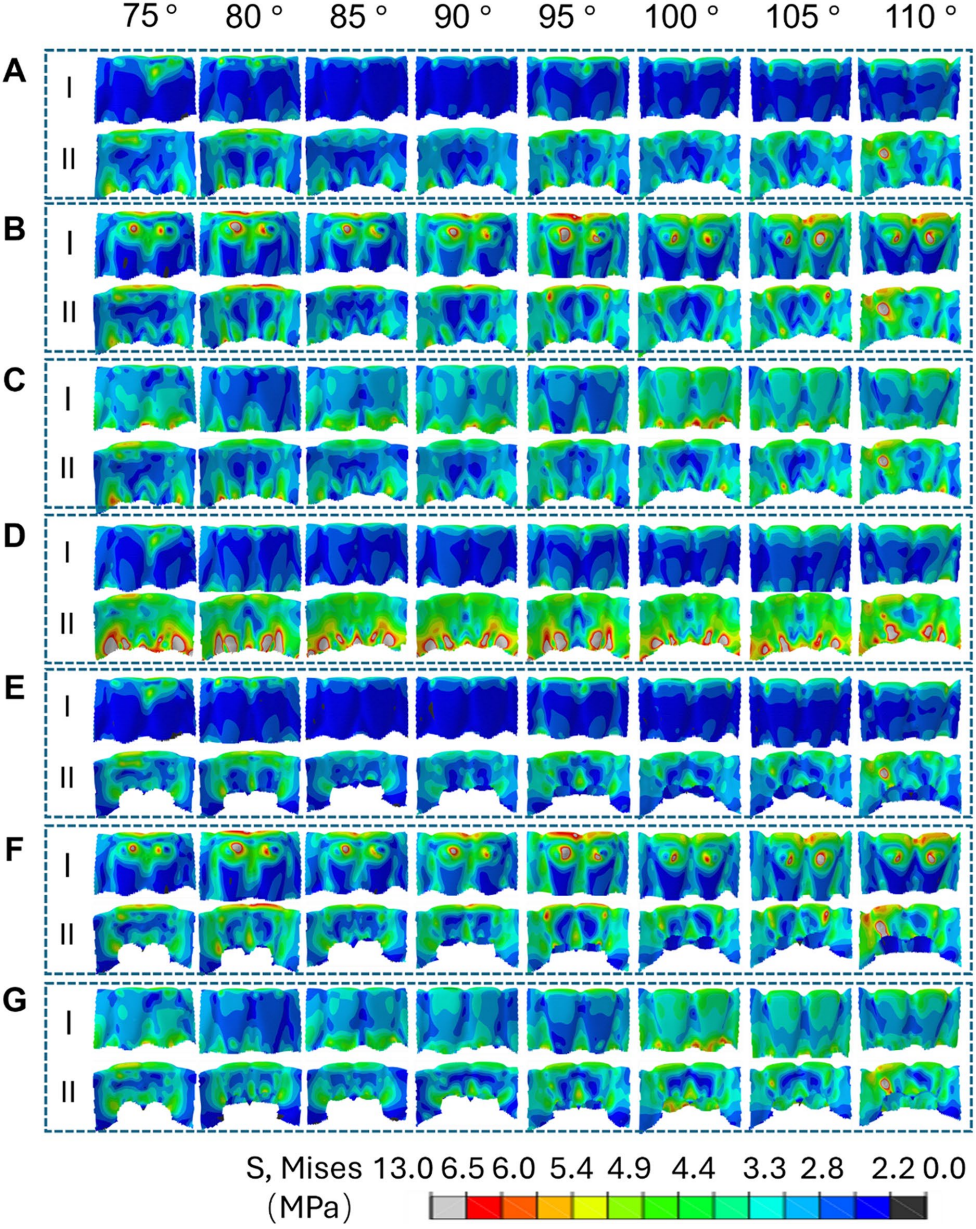
Von Mises stress analysis of CA during intrusion of MAT. Group I represents the labial side, and Group II represents the lingual side. Conventional group (**A**), LIA group (**B**), labial RCR group (**C**), lingual RCR group (**D**), LFEH group (**E**), combination of LFEH and LA group (**F**), and combination of LFEH and labial RCR group (**G**). The bar indicates the Von Mises stress values on the aligner, with red representing high stress and blue representing low stress

Figure 5 illustrates the Von Mises stress analysis of CA during the intrusion of MAT. In comparison with the conventional group (Fig. 5A-I), the LIA group significantly increases the stress on the labial side of the anterior teeth (Fig. 5B-I). When the labial RCR is added, the stress on the labial side also increases, though not as much as with the LA (Fig. 5C-I). The lingual control ridge markedly raises the stress on the lingual side (Fig. 5D-II), while the LFEH group significantly reduces stress on both the lingual and labial sides (Fig. 5E). The combination of LFEH and LA, as well as the use of LA alone, can reduce the stress on the labial side of the anterior teeth (Fig. 5F). Compared to the simple labial RCR, the combination of LFEH and labial RCR decreases the stress on the labial side of the anterior teeth, but increases it when compared to the simple LFEH group (Fig. 5G). Compared to the LFEH group and the combined group with both LFEH and labial RCR, the labial RCR alone increased lingual stress to some extent. However, it was also observed that labial stress increased after the addition of the labial RCR. From the inclination angle analysis, the greater the IMPA, the higher the stress generated on the lingual side (Fig. 5E and G). Comparing the labial RCR group, the LFEH group, and the combination of both, it was found that the labial RCR produced significant stress at the lower edge of the aligner at certain angles. In contrast, the combination of the LFEH and the labial RCR reduced this stress due to the LFEH, leading to less stress concentration than in the labial RCR group (Fig. 5C and G).

### Analysis of contact forces exerted by CA on the labial and lingual sides of MAT during intrusion

**Fig. 6:**
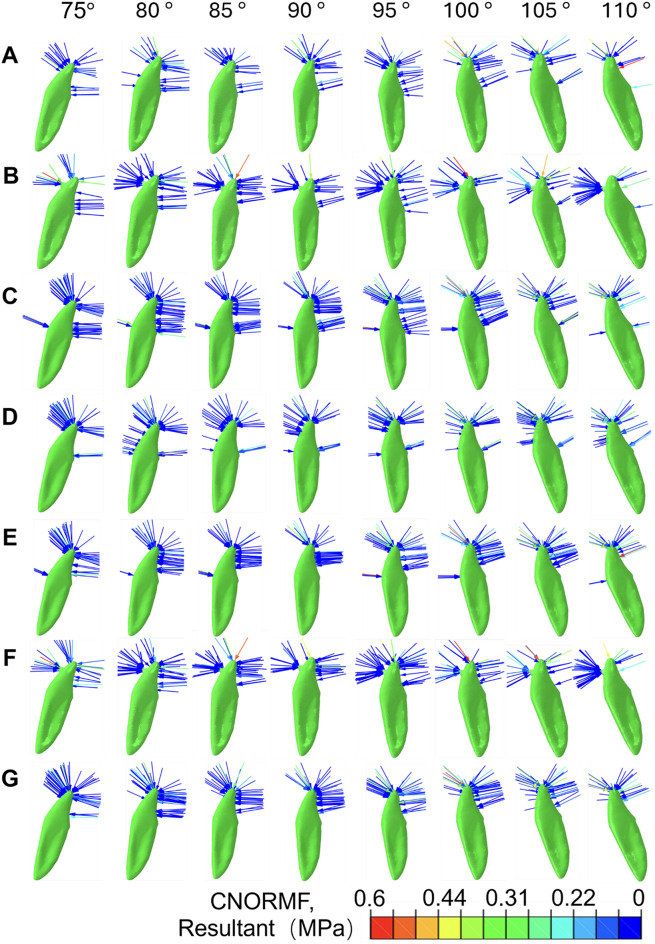
Contact force analysis of different LIA groups (**A**-**G**) under various labial inclination angles (75°, 80°, 85°, 90°, 95°, 100°, 105°, 110°). Conventional group (**A**), LIA group (**B**), labial RCR group (**C**), lingual RCR group (**D**), LFEH group (**E**), combination of LFEH and LA group (**F**), and combination of LFEH and labial RCR group (**G**)

The distribution of contact forces in the LIA group under different labial inclinations is illustrated in Fig. 6. In the conventional group, when the IMPA was 80°, 85°, and 90°, a lingual force was observed on the labial side of the incisal edge. As the inclination increased, so did the lingual force. When the IMPA reached 95°, 100°, 105°, and 110°, a labial force appeared on the lingual side of the incisal edge, with the force increasing proportionally to the inclination (Fig. 6A). The force exerted by the LA was primarily located on the labial side, directed toward the lingual side. Compared to the conventional group, the LIA group significantly increased the lingual contact force on the teeth. In the labial RCR group, the force was mainly concentrated at the labial neck of the teeth, also directed lingually (Fig. 6B). This group was able to significantly enhance the lingual contact force (Fig. 6C). In the lingual RCR group, there was a notable concentration of contact force at the lingual cervical margin (Fig. 6D).

The LFEH group effectively reduces the constraints on the lingual side of the tooth. As a result, the contact force at the lingual fossa is significantly reduced, while the force in the labial-lingual direction is notably increased (Fig. 6E). In comparison with the LA group, the combined group of LFEH and LA demonstrates a clear increase in contact force at the attachment site, with the resultant force directed towards the lingual side (Fig. 6E and F). Compared to the simple labial RCR group, it is evident that the lingual contact force on the labial surface disappears in the LFEH group and the labial RCR group. The excavation of the lingual fossa increases the overall lingual contact force on the teeth (Fig. 6C and G). Additionally, compared to the labial RCR group, the LFEH group significantly reduces the lingual contact force on the labial surface, leading to a more concentrated lingual contact force (Fig. 6E and G).

## Discussions

By measuring and analyzing CBCT data before and after the treatment of mandibular ATI using CA in clinical cases, we found that these teeth are prone to undesigned buccal and lingual inclinations, particularly in the labial and lingual directions. Clinical data indicated that the pre-treatment inclination angle of the mandibular incisors was less than 90 degrees, with an average of 69.07 degrees, while the average IMPA after treatment was 67.79 degrees. The distance from the root’s long axis to the lingual ABW was 4.961 mm before treatment and 4.600 mm after treatment. Similarly, the distance from the root’s long axis to the labial ABW was 2.325 mm before treatment and 2.471 mm after treatment. According to paired t-test results, these changes were statistically significant (*P* < 0.01). The teeth exhibited a tendency toward undesigned lingual crown inclination and labial root inclination. The exact cause of this undesigned buccal and lingual inclinations during the intrusion process remains unclear.

Most clinical studies on ATI focus on the effectiveness of intrusion and the strategies used (such as step-by-step or overall intrusion) [14, 20]. However, few studies have examined the undesigned buccal and lingual inclinations that occurs during the intrusion process. Currently, FEA models of mandibular ATI using CA primarily focus on the stress distribution in upright mandibular incisors, without in-depth exploration of varying inclination angles [29, 31–33]. This article hypothesizes that the risk of undesigned labial-lingual movement of the crown and root may be related to the initial labial-lingual inclination of the mandibular incisors, the direction of force applied by the CA, and certain design features of the aligners (such as attachment and RCR). Therefore, this study constructed a three-dimensional FEA model of mandibular incisors with varying inclination angles during ATI using CA, and analyzed the labial-lingual movement patterns of the root and crown to investigate the biomechanical mechanisms underlying this phenomenon.

To improve clinical outcomes, various localized design modifications of CA were implemented, including the LIA group, labial RCR group, lingual RCR group, LFEH group, LFEH with LA group, and LFEH with labial RCR group (Fig. 2E), followed by FEA. A moment refers to the rotational effect generated by a force acting on an object. In orthodontics, the greater the moment exerted on a tooth, the easier it is to alter its rotational state. Therefore, this study utilizes the moment of the mandibular incisors to analyze the movement trends of the teeth (Fig. 3). Additionally, the change in tooth position and displacement cloud maps before and after treatment were used to assess the displacement changes following intrusion (Fig. 4). The design of additional devices in the anterior teeth area influences the stress distribution in this region, so the Von Mises stress distribution on the labial and lingual sides of the anterior teeth area was measured (Fig. 5). CA exert a wrapping force on the crown, and the surface contact force between the tooth and aligner significantly affects the correction outcome [34–37]. Therefore, this study also analyzes the contact force on the crown during mandibular incisor intrusion (Fig. 6).

### Biomechanical mechanism analysis of undesigned labiolingual displacement of the crown during vertical intrusion of MAT with CA

In the conventional group without auxiliary structures, clinical statistical analysis revealed that the initial IMPA was less than 90 degrees, and after treatment, the average IMPA further decreased. This resulted in lingual crown inclination and labial root inclination. The distance between the root’s long axis and the labial ABW was reduced, while the distance to the lingual ABW increased (Fig. 2D-G). Moment analysis showed that teeth with upright positions (IMPA = 90°, 95°) experienced minimal labial-lingual moments during intrusion, while labially inclined teeth (IMPA = 100°, 105°, 110°) exhibited a significant labial-lingual moment during the intrusion process. Similarly, lingually inclined teeth (IMPA = 70°, 80°, 85°) displayed a large lingual moment. The more severe the labial or lingual inclination, the more pronounced this trend became (Fig. 3F). Therefore, the undesigned buccal and lingual inclinations of teeth may be due to the susceptibility of inclined teeth to lingual or labial moments. The FEA model’s displacement cloud diagrams also indicated that significant lingual displacement occurred when the IMPA was 75°, 80°, and 85°. Conversely, when the IMPA was 100°, 105°, and 110°, noticeable labial displacement was observed (Fig. 4A). To further investigate the biomechanical mechanism behind this phenomenon, we conducted Von Mises stress and contact force analyses. The Von Mises stress cloud diagram showed that teeth with an initial lingual inclination (IMPA = 70°) experienced a large labial force, while those with an initial labial inclination (IMPA = 110°) also exhibited a large labial force (Fig. 5A). Contact force analysis revealed that in the conventional group, when the IMPA was 80°, 85°, and 90°, lingual force appeared on the labial side of the incisal edge, with the force increasing as the inclination increased. Conversely, when the IMPA was 95°, 100°, 105°, and 110°, labial force appeared on the lingual side of the incisal edge, with the force also increasing with greater inclination (Fig. 6A).

### Biomechanical Mechanism Analysis in the design of conventional CA attachments

LA are commonly used in CA to enhance the retention between the tooth and the aligner [6, 38]. However, simple LA may not always effectively achieve true vertical intrusion during anterior teeth correction. Moment analysis after adding LA shows an increase in the lingual moment of the teeth (Fig. 3G), indicating that the addition of LIA promotes a tendency for the crown to move toward the lingual side. For the 95° group, the moment shifted from a labial moment in the conventional group to a lingual moment. However, in cases of severe labial inclination (105°, 110°), the change in lingual moment due to the LIA was less significant. Displacement cloud maps further confirmed that the crown tends to tilt more easily toward the lingual side (Fig. 4B). From the force cloud diagram, it is evident that the force on the LA is concentrated on its labial side, with the direction of the force being lingual (Fig. 5B). According to the contact force diagram (Fig. 6B), the LA group exhibits a denser lingual contact force at the attachment compared to the conventional group. FEA results indicate that after the addition of the LA, the force point was not aligned with the horizontal plane of the attachment, leading to increased local misfit between the aligner and the tooth, thereby reducing its intended effectiveness. This study suggests that these results are related to the unique wrapping mechanism of CA, indicating that when mandibular incisors are engaged with the attachment, a non-fitting “degloving” effect occurs between the gingival side of the attachment and the aligner, resulting in crown tilting toward the lingual side. Adjustments to the inclination of the LA slope or localized improvements to the aligner’s design could be considered.

The labial RCR is also an effective method commonly used in CA to control tooth movement [39, 40]. When the labial RCR is added to the aligner, there is an increased tendency for the crown to move toward the labial side. Moment analysis revealed that in the group with IMPA = 85°, the crown shifted labially, while in the conventional group with IMPA = 95°, the same movement was observed. This indicates that the labial RCR can indeed facilitate labial movement of the crown and lingual movement of the root, resulting in a positive moment effect. This effect is particularly notable in cases of severe labial inclination (IMPA = 85°, 90°, 95°, 100°, 105°, 110°), where the labial moment increases during intrusion. In contrast, for cases with severe lingual inclination (IMPA = 75°, 80°), the addition of the labial RCR increases the lingual moment. As shown in Figs. 3H and 5C, the labial RCR group exhibited a significantly higher concentration of stress on the labial side compared to the conventional group. Figure 6C demonstrates that in the labial control group, the contact force at the labial cervical margin was more concentrated than in the conventional group. This indicates that the labial cervical margin increases the force exerted on the tooth toward the lingual side. Clinically, for teeth with an initial lingual inclination, designing labial RCR attachments can help achieve labial inclination during intrusion. However, when the IMPA is 75° or 80°, the labial RCR can only reduce the tendency of lingual crown (labial root) movement but cannot completely reverse it, potentially requiring the support of additional designs.

The lingual RCR is another effective method for controlling tooth movement, commonly used in CA. In the group with the lingual RCR, all cases exhibited a tendency for the crown to move toward the lingual side. This suggests that for teeth with labial inclination, which is often seen in skeletal Class II cases, adding a lingual RCR can help prevent further labial inclination. As shown in Fig. 3I, the labial moment increased significantly compared to the conventional group. The lingual RCR group also showed the largest displacement (Fig. 4D). Figure 5D indicates that the concentration of lingual force increased significantly in the lingual RCR group compared to the conventional group. Additionally, Fig. 6D illustrates that the contact force at the lingual cervical margin was more concentrated in the lingual RCR group. This indicates that the lingual cervical margin increases the force exerted on the tooth toward the labial side. However, when a patient’s MAT are inclined and the labial alveolar bone is thin, the use of a lingual RCR should be approached with caution. Otherwise, it may lead to crown lingual inclination and root movement toward the labial side, potentially breaching the cortical bone. In patients with skeletal Class III, the mandibular incisors often exhibit compensatory lingual inclination. In these cases, orthodontic treatment typically aims to preserve the lingual inclination of the mandibular incisors. Therefore, the use of a lingual RCR should be avoided, and in situ intrusion should be maintained as much as possible.

### Analysis of the Biomechanical mechanisms in the design of New CA attachments

In the LFEH group, the lingual moment was significantly reduced, while the labial moment increased compared to the conventional group (Fig. 3J). The displacement in the LFEH group was also smaller than that in the conventional group (Fig. 4E). This may be due to the reduction of the lingual side coverage by the aligner in the LFEH group, which decreases resistance and increases the labial moment on the tooth. The force cloud diagram shows that by excavating the lingual fossa, the lingual side force is reduced, and the labial side force is similarly decreased (Fig. 5E). According to the contact force diagram (Fig. 6B), the LFEH group, compared to the conventional group, exhibits a denser lingual contact force on the labial side due to the reduction in counteracting force on the lingual side (Fig. 6E). This effect was not pronounced in the IMPA = 90° group.

In the LFEH group combined with the LA group, the lingual moment was significantly reduced, and the labial moment increased compared to both the conventional group and the LFEH group alone (Fig. 3K). In the group with only LIA, for cases of severe labial inclination (IMPA = 105°, 110°), the addition of LIA did not significantly alter the lingual moment. However, a significant change in the lingual moment was observed after the addition of the LFEH (Fig. 3K).

From the force cloud diagram, it is clear that, compared to the LFEH group, the labial force increased due to the influence of the LA, and the lingual force also increased (Fig. 5F). The contact force diagram shows that the combination of the LFEH and LA significantly increased the dense lingual contact force on the labial side, compared to the LFEH group alone (Fig. 6F). When comparing Fig. 6E with Fig. 6F, it is evident that the addition of the LA generates more contact forces at the attachment, and the resultant force is directed lingually. As a result, at the same angle, teeth with the LA show greater lingual displacement. Additionally, it was observed that the presence of the LA significantly increased the contact force at the attachment site, which weakened the lingual contact force on the labial surface generated by the excavation group. This indicates that the impact of the attachment on tooth movement is much greater than that of the excavation.

Compared to the conventional group, the lingual moment was significantly reduced, while the labial moment increased in both the LFEH group and the labial RCR group. Additionally, the labial moment was further increased in the labial RCR group compared to the LFEH group (Fig. 3L). In the single labial RCR group, under conditions of severe lingual inclination (IMPA = 75°, 80°), the addition of the labial RCR increased the lingual moment (Fig. 3H). The inclusion of the LFEH significantly increased the labial moment (Fig. 3L). As shown in the force cloud diagram, compared to the LFEH group, the labial force increased due to the influence of the LA, with an increase in lingual force as well (Fig. 5F). The contact force diagram demonstrates that, compared to the LFEH group alone, the combination of the LFEH and LA led to a denser concentration of lingual contact force on the labial side, influenced by the LA (Fig. 6F).

## Limitations

In this study, we have conducted the clinical statistical analysis and FEA. Due to the influence of individual differences on clinical data, there may be significant differences in treatment responses among different patients, making it difficult to draw universal and regular conclusions. Although FEA models can summarize regular results under different loading conditions, they may not fully simulate the actual clinical situation. During orthodontic treatment, tooth movement involves the remodeling and reconstruction of the alveolar bone. However, this study conducted a FEA only on the teeth and aligners, lacking research related to the alveolar bone. Nonetheless, this does not affect the overall trend of the experimental results, though more detailed research on this aspect will be needed in future experiments. Additionally, due to space constraints, this study focuses solely on the left mandibular central incisor as a representative for comprehensive analysis of the biomechanical mechanism of tooth intrusion, without analyzing other teeth. A more comprehensive analysis of other teeth will be addressed in future studies.

## Conclusion

During the vertical intrusion of MAT using CA, undesigned labial-lingual displacement of the crown may occur, potentially leading to serious complications. This study proposed an innovative design, the LFEH, and conducted FEA on MAT to analyze the biomechanical changes. The results indicate that the lingual RCR increases the lingual movement tendency of the crown, while the LFEH promotes labial movement. These findings provide valuable guidance for achieving true vertical intrusion of MAT in clinical practice. In clinical treatment, when the crown is inclined lingually, the LIA can be designed to enhance the lingual inclination and intrusion of the teeth. However, when the crown is inclined labially, it necessary to incorporate a LFEH. The labial RCR can enhance the labial movement of the crown, but for teeth with an initial lingual inclination, a LFEH is required. Therefore, during the intrusion of MAT, a comprehensive design that includes LIA, lingual RCR, labial RCR, and LFEH is essential to ensure true vertical intrusion.

## Data Availability

No datasets were generated or analysed during the current study.
